# Racial differences in treatment adherence and response to acupuncture and cognitive behavioral therapy for insomnia among Black and White cancer survivors

**DOI:** 10.1002/cam4.7344

**Published:** 2024-08-19

**Authors:** Kevin T. Liou, Sheila N. Garland, Salimah H. Meghani, Nadia M. Kaye, Embree Thompson, Q. Susan Li, Jun J. Mao

**Affiliations:** ^1^ Department of Medicine, Integrative Medicine Service Memorial Sloan Kettering Cancer Center New York New York USA; ^2^ Department of Psychology and Oncology Memorial University of Newfoundland St. John's Newfoundland and Labrador Canada; ^3^ Department of Biobehavioral Health Sciences, School of Nursing University of Pennsylvania Philadelphia Pennsylvania USA; ^4^ Stuyvesant High School New York New York USA; ^5^ Weill Cornell Medical College New York New York USA

**Keywords:** acupuncture, cancer survivorship, cognitive behavioral therapy, insomnia, oncology, racial disparities, sleep health

## Abstract

**Background:**

Racial disparities in sleep are well‐documented. However, evidence‐based options for addressing these disparities are lacking in cancer populations. To inform future research on sleep interventions, this study aims to understand racial differences in treatment responses to acupuncture and cognitive behavioral therapy for insomnia (CBT‐I) among Black and White cancer survivors.

**Methods:**

We conducted a secondary analysis of a comparative effectiveness trial evaluating acupuncture versus CBT‐I for insomnia in cancer survivors. We compared insomnia severity, sleep characteristics, and co‐morbid symptoms, as well as treatment attitudes, adherence, and responses among Black and White participants.

**Results:**

Among 156 cancer survivors (28% Black), Black survivors reported poorer sleep quality, longer sleep onset latency, and higher pain at baseline, compared to White survivors (all *p* < 0.05). Black survivors demonstrated lower adherence to CBT‐I than White survivors (61.5% vs. 88.5%, *p* = 0.006), but their treatment response to CBT‐I was similar to white survivors. Black survivors had similar adherence to acupuncture as white survivors (82.3% vs. 93.4%, *p* = 0.16), but they had greater reduction in insomnia severity with acupuncture (−3.0 points, 95% CI −5.4 to 0.4, *p* = 0.02).

**Conclusion:**

This study identified racial differences in sleep characteristics, as well as treatment adherence and responses to CBT‐I and acupuncture. To address racial disparities in sleep health, future research should focus on improving CBT‐I adherence and confirming the effectiveness of acupuncture in Black cancer survivors.

## 1 INTRODUCTION

Insomnia is a prevalent, disruptive, and costly condition that affects up to two‐thirds of cancer survivors.^1^, ^2^ Poor sleep health, including excessively short or long sleep durations, has been linked to increased cancer risk and cancer‐related survival.^3^, ^4^, ^5^, ^6^, ^7^, ^8^ A growing body of research has demonstrated that Black individuals experience shorter, lighter, and more fragmented sleep than their white counterparts.^9^ Therefore, improving sleep health has been identified as a potential approach to reduce racial disparities in cancer‐related outcomes and promote more equitable cancer care.^10^, ^11^

While racial disparities in sleep are well‐documented, there is a paucity of research on effective interventions to improve sleep health in Black cancer patients.^9^, ^12^ Prior studies have demonstrated that certain health interventions found to be effective for white individuals may not be as acceptable or effective for Black individuals.^13^, ^14^, ^15^ Acupuncture and cognitive‐behavioral therapy for insomnia (CBT‐I) are evidence‐based, non‐pharmacological treatments that have demonstrated effectiveness for insomnia in cancer survivors.^16^, ^17^ However, due to under‐representation of Black individuals in these clinical trials, their adherence and responses to these two interventions have not been well‐studied.

The aim of the current study is to evaluate racial differences between Black and White cancer survivors with regards to insomnia severity, sleep characteristics, and co‐morbid symptoms, as well as treatment attitudes, adherence, and responses to acupuncture and CBT‐I, using data from a comparative effectiveness trial completed by our group.^18^ The findings are intended to help inform future research on evidence‐based interventions to reduce sleep health disparities in oncology.

## 2 METHODS

### 2.1 Design, setting, and participants of a comparative effectiveness trial

We conducted the sub‐group analysis of a randomized clinical trial that examined the comparative effectiveness of acupuncture versus CBT‐I in cancer survivors with insomnia. The parent trial protocol and primary findings have been published previously.^18^, ^19^ Briefly, the trial was conducted at two academic cancer centers from February 2015 to July 2017. Inclusion criteria were: (1) English‐speaking adults with a cancer diagnosis of any type or stage; (2) completion of cancer treatment with surgery, chemotherapy, and/or radiotherapy at least 1 month before enrollment; (3) score ≥8 on the Insomnia Severity Index;^20^ and (4) insomnia disorder criteria as defined by the Diagnostic and Statistical Manual of Mental disorders, 5th Edition DSM‐(5).^21^ Exclusion criteria were: (1) other inadequately treated sleep disorders; (2) prior insomnia treatment with acupuncture or CBT‐I; (3) inadequately treated psychiatric disorder; and (4) shift work that interferes with establishing regular sleep schedule. Eligible patients completed informed consent and were randomized in a 1:1 ratio to one of the two interventions using permuted block randomization with full allocation concealment. Study investigators were blinded to treatment assignment. The study was approved by the institutional review boards at the University of Pennsylvania and Memorial Sloan Kettering Cancer Center. Through dedicated efforts in patient engagement and outreach,^19^, ^22^ the parent trial enrolled a diverse study population with 27% of trial participants identifying as Black, thereby allowing for exploratory sub‐group analyses. In the current study, we conducted a secondary analysis restricted to Black and White trial participants.

### 2.2 Interventions

Acupuncture is a non‐pharmacologic modality from Traditional Chinese Medicine (TCM) in which practitioners insert thin, sterile, single‐use, metallic needles into the body surface to achieve therapeutic effects.^23^ In this study, licensed acupuncturists with over 10 years of experience delivered the intervention using a manualized protocol. The protocol included standardized points to address insomnia and supplementary points to treat co‐morbid symptoms (e.g., anxiety, pain). Participants received acupuncture treatments twice per week for the first two weeks, followed by weekly for the six remaining weeks, equaling a total of 10 treatments.

CBT‐I is a manualized multi‐component intervention that includes sleep restriction, stimulus control, cognitive restructuring, relaxation training, and sleep hygiene education.^24^ Four licensed therapists and five psychology trainees delivered the CBT‐I intervention. Patients received five weekly sessions followed by two bi‐weekly sessions for a total of seven sessions over 8 weeks.

### 2.3 Outcomes

Insomnia severity, sleep characteristics, co‐morbid symptoms (pain, fatigue, anxiety, depression), and health‐related quality of life were assessed using validated patient‐reported outcomes. The primary outcome of the parent comparative effectiveness trial was insomnia severity as assessed by the Insomnia Severity Index (ISI).^25^ Sleep quality was assessed with the Pittsburgh Sleep Quality Index (PSQI).^26^ Sleep onset latency (time to fall asleep), wake after sleep onset (amount of time spent awake during the night), total sleep time, and sleep efficiency (percentage of time in bed actually sleeping) was calculated using the Consensus Sleep Diary (CSD).^27^ Pain severity was assessed using the Brief Pain Inventory (BPI).^28^ Fatigue was assessed using the Multidimensional Fatigue Symptom Inventory‐Short Form (MFSI‐SF).^29^ Anxiety and depression was assessed using the Hospital Anxiety and Depression Scale.^30^ Physical health‐ and mental health‐related quality of life were assessed using the PROMIS‐Global Health.^31^

To assess views and attitudes towards acupuncture and CBT‐I, we used the Treatment Acceptability and Preference (TAP) scale.^32^ A patient advisory panel collaborated with the research team to develop brief descriptions of acupuncture and CBT‐I, including intervention components, treatment schedule (i.e., number of sessions, frequency, and mode of delivery), potential benefits and risks, and role of the treatment provider.^19^ This information was presented to patients at a 6th grade reading level before the TAP scale was administered. The TAP scale asks patients about their views towards four attributes of the interventions: (1) appropriateness (i.e., does the treatment seem logical for addressing insomnia?); (2) suitability (i.e., does the treatment fit a patient's lifestyle); (3) effectiveness (i.e., is this treatment effective for insomnia?); and (4) convenience (i.e., is the patient willing to apply and adhere to the treatment?). Patients rated each attribute on a 5‐point scale indicating level of agreement: “not at all” (0), “somewhat” (1), “neutral” (2), “very” (3), and “very much” (4). A total score is calculated as a mean of the four attribute scores to reflect overall treatment acceptability; higher scores indicate that patients perceive the intervention as more appropriate, suitable, effective, and convenient. The TAP scale has demonstrated internal consistency, reliability, and factorial validity.^32^ We administered the TAP scale at baseline prior to randomization and receipt of the treatments.

We also assessed treatment adherence by tracking the number of sessions attended. Patients were considered adherent to treatments if they attended ≥80% of the treatment sessions.

### 2.4 Statistical analyses

We used Student's *t*‐tests and chi‐squared tests to evaluate racial differences in insomnia severity, sleep characteristics, co‐morbid symptoms, TAP scale scores, and treatment adherence. To evaluate the impact of race on treatment response, we developed a multivariable linear regression model, with the total ISI score at Week 8 (end of treatment) as the dependent variable, race as the independent variable (White race as the reference), and baseline ISI score as the covariate. We also repeated the regression model for other sleep outcomes, that is PSQI score and all CSD variables. The regression analyses were performed in each treatment group separately. The sample size was pre‐determined by the parent trial.^18^ All analyses were post‐hoc. All statistical tests were two‐sided. Statistical significance was set at *p* < 0.05. All statistical analyses were conducted using STATA (version 15.0; STATA Corporation, College Station, TX).

## 3 RESULTS

Participant characteristics are summarized in Table 1. Among the 160 patients enrolled in the parent trial, 43 were Black and 113 were White. Of these 156 patients, 78 were assigned to receive acupuncture and 78 were assigned to receive CBT‐I.

**TABLE 1:** Patient characteristics.

	Total (*N* = 156)		Acupuncture (*N* = 78)		CBT‐I (*N* = 78)	
	*N*	%	*N*	%	*N*	%
Age (mean, SD), years	61.4 (11.7)		62.3 (11.3)		60.5 (12.1)	
Race						
White	113	72.4	61	78.2	52	66.7
Black	43	27.6	17	21.8	26	33.3
Gender						
Male	67	43.0	36	46.2	31	39.7
Female	89	57.0	42	53.8	47	60.3
Education						
Some college or less	45	28.9	24	30.8	21	26.9
College graduate or higher degree	111	71.2	54	69.2	57	73.1
Marital status						
Married or with partner	79	50.6	38	48.7	41	52.6
Not living with partner	77	49.4	40	51.3	37	47.4
Cancer type^a^						
Breast	50	32.1	24	30.8	26	33.3
Prostate	34	21.8	18	23.1	16	20.5
Other	72	46.2	36	46.2	36	46.2
Cancer stage						
Stage 0	5	3.2	2	2.6	3	3.9
Stage I	69	44.2	35	44.9	34	43.6
Stage II	38	24.4	18	23.1	20	25.6
Stage III	28	18.0	14	18.0	14	18.0
Stage IV	13	8.3	7	9.0	6	7.7
Unknown	3	1.9	2	2.6	1	1.3
Years since cancer diagnosis, mean (SD)	6.0 (5.3)		6.3 (4.8)		5.7 (5.7)	
Years since insomnia onset, mean (SD)	9.2 (9.2)		10.0 (9.6)		8.5 (8.7)	
Insomnia Severity Index Total, mean (SD)	18.1 (4.3)		17.7 (4.1)		18.4 (4.4)	
Pittsburgh Sleep Quality Index total, mean (SD)	11.9 (3.4)		11.8 (3.2)		12.0 (3.6)	
Sleep diary variables						
Minutes sleep onset latency, mean (SD)	38.6 (48.1)		30.7 (27.1)		47.2 (62.6)	
Minutes awake after sleep onset, mean (SD)	55.9 (40.7)		57.8 (39.3)		53.9 (42.4)	
Minutes total sleep time, mean (SD)	342.4 (89.5)		346.8 (83.1)		337.6 (96.4)	
Sleep efficiency percentage, mean (SD)	0.73 (0.16)		0.73 (0.14)		0.72 (0.18)	
Brief Pain Inventory, pain severity, mean (SD)	2.3 (2.2)		2.1 (2.0)		2.4 (2.4)	
Multidimensional Fatigue Symptom Inventory—short form total, mean (SD)	20.7 (21.8)		19.6 (21.2)		21.8 (22.5)	
Hospital Anxiety and Depression Scale						
Anxiety, mean (SD)	7.8 (4.2)		7.8 (4.1)		7.7 (4.3)	
Depression, mean (SD)	4.8 (3.2)		4.6 (3.1)		5.1 (3.4)	
PROMIS Global health						
Physical health	44.4 (8.5)		45.2 (8.2)		43.5 (8.7)	
Mental health	44.7 (8.1)		45.1 (7.9)		44.2 (8.2)	

^a^ Other cancer types include colorectal, head/neck, hematologic, gynecologic, skin, lung, other gastrointestinal, other genitourinary, and >1 cancer type.

Baseline sleep characteristics and co‐morbid symptoms differed by race (Table 2). White survivors experienced insomnia symptoms for a longer duration (10.4 [SD 9.6] vs. 6.4 [SD 7.3] years, *p* = 0.01), whereas Black survivors reported poorer baseline sleep quality (PSQI total score, 13.4 [SD 3.5] vs. 11.4 [SD 3.2], *p* < 0.001), longer sleep onset latency (58.3 [SD 53.4] vs. 32.0 [SD 44.4] min, *p* = 0.004), higher pain severity (BPI score, 3.0 [SD 2.8] vs. 2.0 [SD 1.9], *p* = 0.02), and poorer physical health‐related quality of life (PROMIS 41.7 [SD 9.5] vs. 45.4 [SD 7.9], *p* = 0.02).

**TABLE 2:** Baseline sleep characteristics and co‐morbid symptoms by race.

	White	Black	*p* value
Years since insomnia onset, mean (SD)	10.4 (9.6)	6.4 (7.3)	0.014
Insomnia Severity Index Total, mean (SD)	17.7 (4.2)	18.9 (4.3)	0.10
Pittsburgh Sleep Quality Index Total, mean (SD)	11.4 (3.2)	13.4 (3.5)	<0.001
Sleep diary variables			
Minutes sleep onset latency, mean (SD)	32.0 (44.4)	58.3 (53.4)	0.0042
Minutes awake after sleep onset, mean (SD)	55.3 (35.9)	57.6 (52.8)	0.77
Minutes total sleep time, mean (SD)	348.7 (86.1)	323.9 (97.8)	0.15
Sleep efficiency percentage, mean (SD)	0.74 (0.15)	0.69 (0.17)	0.068
Brief Pain Inventory, Pain Severity, mean (SD)	2.0 (1.9)	3.0 (2.8)	0.015
Multidimensional Fatigue Symptom Inventory—Short Form Total, mean (SD)	20.2 (22.1)	22.2 (21.3)	0.60
Hospital Anxiety and Depression Scale			
Anxiety, mean (SD)	7.8 (4.3)	7.5 (4.0)	0.70
Depression, mean (SD)	4.8 (3.1)	4.8 (3.5)	0.92
PROMIS global health			
Physical health	45.4 (7.9)	41.7 (9.5)	0.015
Mental health	45.2 (7.8)	43.3 (8.6)	0.19

Black and White survivors had no significant differences in their views about the appropriateness, suitability, and effectiveness of acupuncture and CBT‐I. However, compared with White survivors, Black survivors viewed both interventions as less convenient. Regarding the convenience of acupuncture, 74.4% of Black survivors responded “very” or “very much” to the TAP convenience item (i.e., willing to apply and adhere to the treatment), compared with 90.3% of White survivors (*p* = 0.03). Regarding the convenience of CBT‐I, 57.1% of Black survivors responded “very” or “very much” to the TAP convenience item, compared with 79.6% of white survivors (*p* = 0.04).

Treatment adherence differed by race. With respect to acupuncture, 82.3% of Black survivors adhered to treatments, compared to 93.4% of White survivors (*p* = 0.16). With respect to CBT‐I, 61.5% of Black survivors adhered to treatments, compared to 88.5% of white survivors (*p* = 0.006).

In multivariable linear regression analyses of acupuncture recipients, Black race was associated with a greater post‐treatment reduction in the ISI score (−3.0, 95% CI −5.4 to −0.5, *p* = 0.02) and minutes awake after sleep onset (−20.0, 95% CI −36.6 to −3.3, *p* = 0.02), after adjusting for baseline ISI score and baseline minutes awake after sleep onset, respectively. By contrast, when CBT‐I recipients were evaluated in the multivariable linear regression model, race was not significantly associated with any post‐treatment sleep outcomes, after adjusting for baseline scores.

## 4 DISCUSSION

Racial disparities in sleep represent a significant public health concern,^9^, ^33^ and growing research has identified sleep as a promising intervention target to reduce racial inequities in cancer and other chronic diseases.^11^ In this secondary analysis of a comparative effectiveness trial with over 25% of trial participants who identified as Black, we observed racial differences in sleep characteristics, as well as treatment adherence and responses, which could potentially be leveraged to improve sleep interventions for Black cancer populations.

Our study revealed that Black survivors had lower rates of adherence to CBT‐I but similar rates of adherence to acupuncture, compared to their white counterparts. Growing research has demonstrated that neighborhood characteristics (e.g., crime rate, disorder) or home environment conditions (e.g., broken windows) may contribute to racial disparities in sleep among Black populations.^34^, ^35^, ^36^, ^37^ Given that CBT‐I involves techniques that must be applied in everyday settings and home environments, it is possible that some individuals who live in unsafe neighborhoods or poor housing conditions may find it challenging to adhere to CBT‐I. By contrast, not only did more Black survivors view acupuncture as convenient, but they also demonstrated higher rates of adherence to acupuncture than to CBT‐I. While many acupuncturists counsel patients on lifestyle modification in addition to administering the needling techniques, patients may view acupuncture as primarily a receptive therapy in contrast to CBT‐I, which requires more active participation and application of skills by patients. This receptive nature of the intervention may have contributed to perceptions of acupuncture as convenient. However, the underlying reasons for higher acupuncture adherence among Black survivors still requires further exploration in future research. This new understanding could potentially be applied to other sleep interventions to address barriers to adherence.

Despite lower adherence to CBT‐I, Black participants experienced similar treatment response to CBT‐I relative to their white counterparts. Given that CBT‐I is a skill‐based intervention, it is possible that participants were able to learn helpful techniques that they could immediately apply in daily life, even if they did not complete all the remaining sessions. These findings suggest that greater focus should be placed on increasing access to CBT‐I and removing barriers to initiating treatment among Black patients.^38^ There are also recent efforts to tailor CBT‐I to the diverse cultural, social, and environmental contexts of Black communities with the goal of improving engagement, adherence, and treatment outcomes among Black patients.^39^ For example, visual representation in CBT‐I program materials and inclusion of race‐concordant providers were identified as important components that enable Black cancer survivors to see themselves in the intervention. While not specific to cancer survivors, a systematic review examined RCTs of culturally adapted interventions for sleep‐related conditions and found that culturally adapted interventions produce greater benefits.^40^ These findings were supported by a recent 3‐arm RCT comparing a stakeholder‐informed, culturally tailored CBT‐I program to an standard CBT‐I intervention or a patient education control group among Black women.^41^ While both CBT‐I interventions significantly reduced insomnia compared to the control group, significantly more women completed the culturally tailored version than the standard intervention. Completion of the interventions was associated with greater improvements in sleep outcomes. Adopting this culturally tailored approach to CBT‐I may help to further reduce racial disparities in sleep health.

Of note, acupuncture produced greater reduction in insomnia severity and minutes awake after sleep onset among Black survivors compared with White survivors. Although the reasons for this differential response to acupuncture require further investigation, it is possible that Black patients have a unique insomnia phenotype better suited for acupuncture.^42^, ^43^ Consistent with other research demonstrating racial disparities in pain and sleep outcomes,^9^, ^12^, ^44^, ^45^ the Black survivors in our study reported higher pain severity and poorer physical health‐related quality of life, as well as longer sleep onset latency and poorer sleep quality. Pain and sleep disturbances are known to have a bi‐directional relationship,^46^, ^47^ suggesting that each symptom could potentially exacerbate or perpetuate the other if both are not adequately treated. Research from our group has shown that acupuncture not only can jointly treat pain and sleep disturbances,^48^ but also may produce improvements in insomnia by reducing co‐morbid pain,^49^ which is a prevalent symptom in cancer survivors. As such, acupuncture may represent an optimal treatment approach for Black survivors with co‐occurring insomnia and pain, particularly those who are unable to pursue separate treatments for each health issue due to time and financial toxicities or other barriers.^50^, ^51^

Our findings must be considered in the context of several limitations. We conducted a post‐hoc sub‐group analysis of a parent comparative effectiveness trial, so the findings are hypothesis‐generating rather than confirmatory. Due to limited enrollment of other racial/ethnic groups, this study included only Black and White trial participants. Future studies should examine other racial and ethnic groups to provide a more comprehensive understanding of sleep health disparities. The parent trial was also conducted before the widespread adoption of telehealth during the COVID‐19 pandemic. Since CBT‐I, unlike acupuncture, can be delivered remotely, perceptions and attitudes towards these two modalities may have changed. Finally, most trial participants were relatively well‐educated and receiving care at a tertiary academic cancer center, so the generalizability of our findings may be limited.

Despite these limitations, this study is one of the first to document racial differences in treatment adherence and response to acupuncture and CBT‐I among Black and White cancer survivors. Compared to their white counterparts, Black cancer survivors had similar improvements in sleep with CBT‐I but experienced greater improvements with acupuncture, suggesting that both are promising tools to address sleep disparities in this population. Future research should build on these findings and focus on improving CBT‐I adherence and confirming acupuncture effectiveness among Black cancer survivors.

## AUTHOR CONTRIBUTIONS

**Kevin T. Liou:** Conceptualization (equal); investigation (equal); supervision (lead); writing – original draft (lead); writing – review and editing (equal). **Sheila N. Garland:** Investigation (equal); writing – review and editing (equal). **Salimah H. Meghani:** Investigation (supporting); writing – review and editing (equal). **Nadia M. Kaye:** Investigation (supporting); writing – review and editing (supporting). **Embree Thompson:** Investigation (supporting); writing – review and editing (supporting). **Q. Susan Li:** Data curation (lead); formal analysis (lead); writing – review and editing (supporting). **Jun J. Mao:** Conceptualization (equal); funding acquisition (lead); resources (lead); supervision (equal); writing – review and editing (supporting).

## FUNDING INFORMATION

Research reported in this paper was funded in part by a Patient‐Centered Outcomes Research Institute (PCORI) Award (CER‐1403‐14292). This manuscript is also supported in part by a grant from the National Institutes of Health/National Cancer Institute Cancer Center (P30 CA008748) and the Translational Research and Integrative Medicine Fund at Memorial Sloan Kettering Cancer Center. Dr. Liou is supported in part by the National Cancer Institute (1K08CA266927‐01A1). Dr. Meghani is supported in part by the National Cancer Institute (R01CA270483; U01CA286811) and by the National Institute of Nursing Research (R01NR017853). Dr. Mao is supported in part by the National Cancer Institute (R01CA240417). The statements presented in this article are solely the responsibility of the authors and do not necessarily represent the views of the Patient‐Centered Outcomes Research Institute (PCORI), its Board of Governors, or its Methodology Committee.

## CONFLICT OF INTEREST STATEMENT

Dr. Mao reports grants from Tibet CheeZheng Tibetan Medicine Co Ltd outside the submitted work. All other authors declare that the research was conducted in the absence of any commercial or financial relationships that could be construed as a potential conflict of interest.

## ETHICS STATEMENT

This study was approved by the institutional review boards, and all participants provided written informed consent.

## PRECIS

Black cancer survivors are drastically under‐represented in sleep intervention trials. In this secondary analysis of a comparative effectiveness trial, Black cancer survivors demonstrated lower adherence to cognitive behavioral therapy for insomnia and greater reduction in insomnia severity with acupuncture.

## PERMISSION TO REPRODUCE MATERIAL FROM OTHER SOURCES

N/A.

## CLINICAL TRIAL REGISTRATION

NCT02356575.

## Data Availability

The data that support the findings of this study are available from the corresponding author upon reasonable request.
